# Supplementary material for: L-Ascorbic Acid Restricts Vibrio cholerae Survival in Various Growth Conditions
Source: Microorganisms. 2024 Feb 29;12(3):492. doi: 10.3390/microorganisms12030492 (PMC10972366; doi:10.3390/microorganisms12030492)
Supplement: Supplementary file 1 [file microorganisms-12-00492-s001.zip › microorganisms-2849244-supplementary.pdf]

microorganisms MDPI

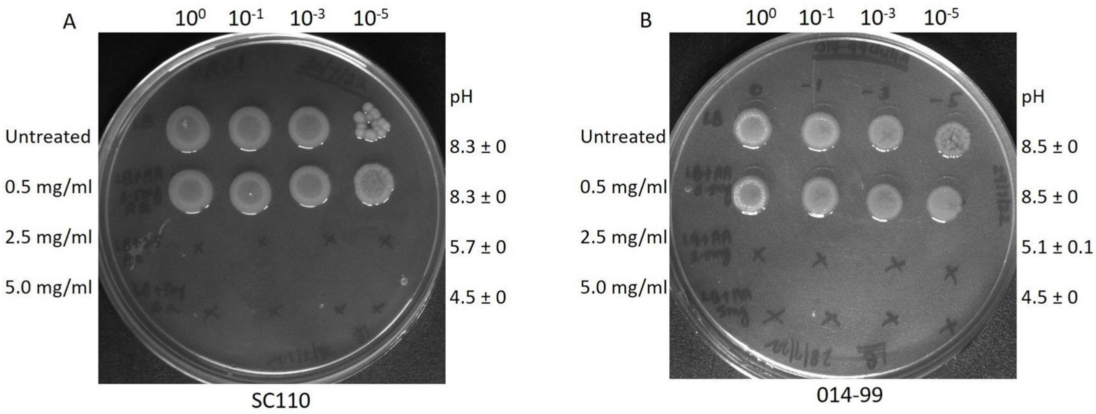

Supplementary Figure S1. Growth of *V. cholerae* strains in presence of L-AA. *V. cholerae* strains SC110 and 014-99 were grown in LB supplemented with increasing concentration of L-ascorbic acid (0, 0.5, 2.5 and 5 mg/ml). Exponential growth phase cultures of both the strains **(A)** SC110 and **(B)** 014-99 were diluted to a starting OD 600_{nm} 0.01 and grown for 10 h. The serially diluted cultures were spotted on solid agar. The plate photographs are a representative of the experiment performed in biological and technical duplicates. The average and standard deviation of the pH values was calculated from the pH measured at the end of each growth assay. pH values and their standard deviations were rounded off.

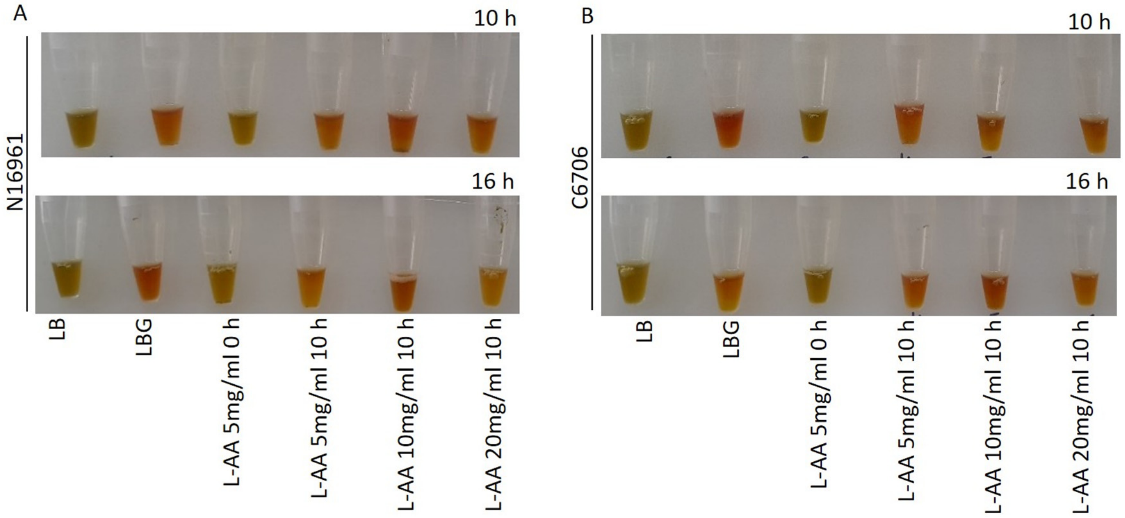

Supplementary Figure S2. Voges–Proskauer (VP) Test of *Vibrio cholerae* El Tor strains to check the production of acetoin. Log phase growth cultures of *V. cholerae* strains N16961 and C6706 were grown in either LB or LB + 1% glucose (LBG). L-AA (5 mg/ml) was added to the LBG flask at the start of the experiment and after 10 h of growth at a concentration of 5, 10 or 20 mg/ml. Acetoin production was checked after 10 h (before adding L-AA) and after 16 h the acetoin production was checked using the VP test for **(A)** *V. cholerae* N16961 and **(B)** *V. cholerae* C6706. The image shown is a representative of the assay performed in biological replicates.
